# Observations on the Growth and Irregularities of Children's Teeth, &c.

**Published:** 1845-07

**Authors:** 


I. Observations on the Growth and Irregularities of Children's Teeth, followed by Remarks and Advice on the Teeth in general. By *W. H. Mortimer.* Small 8vo, pp. 129. Highley, 1845.

II. The Forceps. Vol. I. 1844.

III. On Diseases of the Jaws. By *Richard O'Shaugnessy.* Octavo, pp. 100. Calcutta, 1844.

Although in every department of the Art of Healing the impudent charlatan and uninstructed pretender have managed frequently, by reason of the credulity and ignorance of the public, to obtain a footing at the expense of educated and conscientious practitioners, yet Dental Surgery is their realm *par excellence*, in which they have until of late held an almost undisputed sway. The pain of toothache is torturing, and endurable by few, the remedy by extraction is speedy, simple, and complete, and hence he who has acquired a reputation for manual dexterity is sought for, be his other qualifications what they may. The loss or great decay of teeth produces grievous personal disfigurement, and many other serious evils, and he who promises reparation or prevention is listened to just in proportion as he promises boldly and indiscriminately.

Nor can we feel much surprize that the public is bamboozled in this manner, seeing that the men of upright character and scientific acquirements in this branch of the profession have taken no pains to furnish it with any test of competency, while medical men in general possess little more knowledge upon the subject than do their patients. With the exception of those of Mr. Bell there have not been any courses of lectures delivered upon this part of medical science at any of our schools, at least not until quite recently; and the works of that gentleman and of Mr. Fox are the only ones of a standard character generally accessible. The crowds of patients who flock to the casualty wards of our hospitals, who might, under proper tuition, form an invaluable means of instruction, have, by being consigned over to the tender mercies of raw and unaided pupils, suffered a vast amount more of pain than they need have done, and yet have ministered little or nothing to the improvement of those who have operated upon them. The consequence is, that when the student enters into practice he finds himself, in nine cases out of ten, incompetent to give useful and satisfactory advice as to the prevention or removal of diseases of the teeth, and unable to adopt the best operative procedures fitting for the case when these become unavoidable. This should not be, for although the subdivision of employments in a large town may render the reference of such cases to an accomplished dentist possible, and certainly then advisable, yet the exigencies of country practice do not admit of this. The appointments which of late have been made in several of the hospitals are good signs of improvement, and we hope that speedily no medical school will be without its Professor and Demonstrator of Dental Surgery.

In the mean time we are glad to find the Dentists themselves are putting their shoulders to the wheel, and that, at least the well-educated portion of them, are protesting against the admission of persons to the practice of the art without examination or other guarantee of competency. They justly feel that, until some line of separation is drawn between the advertising quack or mere expert handicraftsman, the *profession* of surgeon-dentist can be scarcely said to exist. To this end they have established a journal, and although, in advocating their just claims, it has proved rather extreme in some of its denunciations and fanciful in some of its suggestions, its general tendency is well worthy of encouragement. Among the recommendations urged by the *Forceps* is the establishment of a College of Dentists; but for this there exists neither *materiel* or necessity. The new medical examining board may be empowered to insist upon peculiar studies, and a particular examination on the part of such as intend to devote themselves to Dentistry; and, while it thus affords the public a guarantee of competency, will offer to the candidate a distinct professional *status* he can in no other way so effectually attain. It is truly stated in the publication in question, that the diploma of the College of Surgeons now held by many dentists, and which has been instrumental in procuring for some of them appointments, is no proof whatever of their skill or attainments in this branch of the profession. In the absence, however, of all tests of qualifications of any kind, it has proved some security to the public, that its holder has taken some pains to inform himself of the structure, functions, and surgical diseases of the human frame, and honorably distinguishes him from those who have not done so. We believe the interests of the public and of the dentist will be best consulted by insisting that his professional education shall, at all events in its preliminary stages, be the same as that of the members of other branches of the medical body.

Mr. Mortimer's little work is chiefly addressed to the public, and contains useful advice, some of which is not to be slighted even by professional persons. It is written in a high and proper tone, reprobating and exposing several of the quackish practices which are so much in vogue. We select a few extracts.

*Lancing the Gums.*—" I stated it will be recollected that the teeth were situated beneath the gums in each jaw, and that it was the pressure of the edge of the tooth on the internal parts of the gum that caused the irritation and pain. Now the depth the tooth has to perforate being considerable, it stands to reason that if the gums are lanced at the first period of inflammation, they will reclose, and as often as the irritation returns, the operation must be repeated; besides which the gums will become harder each time they re-unite. For, although not visible to the eye, a cicatrix is formed, which renders it still more difficult for the tooth to pierce them. The impropriety of performing the operation at this period must be evident, and will account for the *bad practice* complained of. But when the teeth are sufficiently advanced, so as to shew their presence by a white mark, caused by their pressure on the internal parts of the gums; or when a tooth has partially perforated them, then the lancet may, nay *ought* to, be used without delay; for the gums cannot again completely re-close, and the tooth will come through without giving any more pain. In all other cases the lancet should only be used when all other efforts have failed. But if, notwithstanding all our efforts, the inflammation and irritation continue, and cause excessive fever—that frequent returns of the convulsions are apprehended; it must then be left to the sagacity of the medical attendant when he ought to lance the gums: and I have only in these cases to recommend that the wound may be as deep as possible, and directly over the teeth that are supposed to cause the pain."

We have extracted this passage to protest against the doctrine it teaches, and to express our regret that parents may hereafter quote the author's book in enforcement of the prejudices which they so often entertain against this simple yet beneficial operation. It is not the fact that lancing the gum renders the subsequent penetration of the gum by the tooth more difficult, the cicatrix in truth yielding more readily to the action of the absorbents than the original structure. So, too, deep incisions are of vast benefit in relieving the congested and tense condition of the gums long prior to the approach of the tooth to the surface, perhaps months before it is cut, especially when the salivation is not profuse; and surely the various accidents which the author admits depend upon this condition of the gum are most rationally removed by attacking the cause, rather than by first employing other measures.

*Early Cleansing of the Teeth.*—"Children sometimes experience toothache from the first teeth decaying before the others are ready to supply their place; but this is owing, in a great measure, to a want of cleanliness, in not using a tooth-brush. I must here notice one of those prejudices which it is difficult to account for: viz. the obstinacy with which some mothers refuse to allow their children's teeth to be cleaned. I have no hesitation in saying, that as soon as it is possible to make the child open its mouth, the teeth it may have ought to be rubbed. In very early age, the sponge is sufficient. A child ought to be taught to brush his teeth as soon as he is taught to wash his hands and comb his hair; and a habit thus inculcated in early life will never be forgotten. As to the supposition, that the brush will wear the enamel away, it might be argued with equal truth, that the comb will wear the head away."

*First Permanent Teeth.*—After cautioning against the premature removal of the milk teeth, and recommending two amalgams for the purpose of arresting their decay, Mr. Mortimer continues.

"At about six, the first teeth of the second set begin to appear. They are large double teeth, and are situated behind the last milk teeth at each extremity of the upper and lower jaw. When these teeth are perfectly formed, they serve, being placed one over the other, as a support to the jaw, while all the first set are being changed. These teeth, from the early age at which they appear, and from want of attention to cleanliness, almost always decay in early life, and very often shortly after they have made their appearance. During the progress of dentition, no attention is paid to them, under the supposition that they are milk teeth, and that consequently they will be changed: if therefore they decay, no notice is taken of them until they give pain, when the child is taken to the Dentist to have them extracted.

"A *medical* gentleman brought his son to me some time since, begging me to remove one of these teeth that was in a very decayed state. When I complained of the negligence that had allowed his second teeth to decay, instead of having them stopped as soon as any caries appeared, he quite stared, declared it was a first tooth; and it was not until I had produced several anatomical preparations to support my argument, that he could be convinced to the contrary. Another medical gentleman, who had resided for some years out of Europe, brought five of his children to me, and we found that *sixteen* out of *twenty* of these teeth had been removed by the surgeon in whom he had confided where he had been residing; and he assured me he had no doubt but that several of them had been *extracted perfectly sound*. That the public should form an erroneous opinion is pardonable; for, at first sight, it appears strange that Nature should begin by giving us too large double teeth, whereas in the first dentition, she began by the front ones; but one moment's reflection shews us that it is necessary; since, as all the other teeth are to be shed, there would be nothing to keep the jaw in its place, and allow the others to come out regularly. As to my own experience, I can aver, that out of any 100 children that are brought to me of more than 11 years of age, at least 90 have some of these teeth in a decayed state; and 95 out of 100 adults, have lost one or more of them; whereas, had they been properly attended to, they might in all probability have been saved.

"I cannot, therefore, too seriously impress upon my readers the necessity of paying the greatest attention to these teeth, and as soon as any discoloration appears, to have them stopped; and even without that appearance, to have them examined from time to time—for there exists a species of decay not perceptible to the eye, as no discoloration of the teeth takes place, which increases rapidly, and is extremely sensitive when far advanced, but which, if taken in time, may be effectually stopped. Whenever the child is taken to a dentist, always have these teeth probed; but on no account have them extracted, unless they are so far gone that there is no possibility of saving them. There cannot be the least difficulty in distinguishing these teeth from those of the temporary set. I mentioned that there were ten milk teeth in each jaw, and that they were all in their places at about three years of age. The teeth now alluded to appear only at six, and are situated behind the others at the end of the jaw; counting, therefore, five teeth on each side from the centre ones, it is impossible to make any mistake; they are larger, stronger, and yellower than the milk teeth, which have always a blue milky appearance."

There are several other useful cautionary observations respecting the removal of the milk teeth, which are thus summed up. 1. Only remove two teeth at a time, and always the corresponding ones. 2. Never remove the adjoining teeth till those previously extracted are replaced; that is, until they appear at the edge of the gums. 3. If there is not room for the four front teeth, do not remove the canine, but the tooth next it.

The mode of correcting irregularities of the teeth is illustrated by cases and diagrams; but to these we can only refer. We may however extract an additional observation upon the importance of not removing children's teeth too precipitately.

"I cannot continue without referring for a minute to what was said, concerning the necessity of preserving the milk teeth as long as possible. We find that the jaw increases so little in front, that if the least portion of the space destined for the six front teeth is taken up by the adjoining side ones, the canine teeth must come up out of the circle of the other teeth. But if they (the milk canine teeth) are kept in the mouth, there will be plenty of room for the side teeth, which cannot then encroach upon the space allotted to those that are to replace them. The immense increase of the jaw at the side will always permit us to calculate, that there will be room enough for the grinders. Independently of this, we must take into consideration the probability of being obliged to extract some of the first double teeth that appeared at six years, and which would give us more room than we required.

"Never be too hasty in removing a good second tooth to make room for the others. If a tooth appears irregularly, endeavour, if possible, to rectify it by the removal of some of the first teeth, provided it can be done with impunity, and without running the risk of displacing the others. If not allow the jaw to expand, and the teeth to go their own way, until the age of 14; when, if it be found impossible to rectify the deformity by artificial means, then, and only then, the teeth that are in the way should be removed. In all cases where it is found absolutely necessary to extract one or more teeth in either jaw, to make room for others; *never*, under any pretext whatever, allow either of the *six front teeth* to be removed; those next to the canine teeth, called the small grinders, of which there are two on each side, are the teeth to be removed, and none else. It may be said of a Dentist as it is of a Surgeon, that he is clever who performs an operation well, but he is more clever who can cure without an operation. Patience and perseverance are indispensable requisites for those who wish their children to have a good and a healthy set of teeth."

*Cleanliness* is as important (and indeed the only known one) a *preservative* of the teeth in the adult as in the child.

"My advice to persons with regard to cleaning their teeth is this:—Every morning clean the teeth well, both inside and outside, with a brush (neither too hard nor too soft), and some tooth-powder; rinse the mouth afterwards with water, always at a warm temperature, about that of the mouth, into which a little aromatic essence may be poured (Eau de Cologne is as good as any other, unless there is a disease of the gums), to give it an agreeable flavour, taking care to pass the water through the interstices of the teeth (which may be done by closing the mouth) to remove everything that may have escaped the brush. At night pass a soft brush lightly over the teeth; powder is not necessary; and rinse the mouth well. If this treatment will not prevent the teeth from decaying, all the lotions and powders in the universe will only tend to make them go faster. As the teeth have, more or less, a latent predisposition to decay, it is necessary now and then to have them examined. If the least discoloration appears on them, instantly apply to a dentist, that it may be stopped. * * * * * I have often seen teeth that were stopped by the late Mr. Wayte, some 30 or 40 years ago, remain as perfect as the first day. There can be no doubt, therefore, that a tooth properly stopped, will be preserved from any further decay in that spot. It *is* necessary that all the decayed part should be removed; but it is *not* requisite that a quarter of the tooth should be scraped away, as I have been informed is practised by some dentists. This looks like quackery, and is intended to make the operation appear more difficult. But recollect, *procrastination* is fatal to the teeth; if you delay from day to day, when you know there *is* decay, it may happen that, when you go to have them stopped, they will be so far gone that there will be no means of using the necessary pressure without exciting the sensibility of the nerve: in which case the operation cannot be effectually performed, and the loss of the tooth may be the result.

It is not an uncommon occurrence for persons whose teeth are in a very decayed state, whether from constitutional causes, or from the excessive use of calomel, to suppose that nothing can be done to arrest the decay, and that therefore the only plan is to allow them to go on until they are all gone, when recourse may be had to artificial means for supplying their place. It is never too late to attempt to arrest the progress of decay in the teeth. Some, it is true, may be beyond the reach of art, but I never remember to have seen a case in which all the teeth were in the same advanced state. Decay invariably attacks the teeth progressively, and may, as I have before stated, be always arrested before it has penetrated the cavity that contains the nerve. The excessive use (I may say abuse) of calomel causes the greatest havoc to the teeth, and when it has been given in large quantities, medical men ought never to forget to urge upon their patients the necessity of having them attended to as soon as their recovery will admit of it."

*Filing the Teeth,* for the removal of the carious part, so generally practised by dentists, is entirely disapproved of by the author.

"The only reason I shall here give for not filing the teeth is this. If the sides of the orifice are removed by the file, and the operation does not arrest the progress of the decay, you then can never stop the tooth afterwards; and in the course of a few years it will become so discoloured that it must be extracted. It will be asked how are we to stop a tooth that is decayed at the side, when the proximity of the adjoining tooth is such that there is no possibility of getting at it? Take a thin piece of Indian-rubber, place it between them, so that it shall fit tight, and the teeth will separate by degrees. It must be removed every morning, and a larger piece put in its place, and in three days there will be sufficient space to allow the decayed part to be removed, and the tooth to be effectually stopped. As soon as the operation is concluded, and the Indian-rubber removed, the teeth will, in 24 hours, be in their primitive position. This applies to the six front teeth. For the side ones, drill a hole over the decay, enlarge it sufficiently to work the decay away, and stop it."

The opinion that *Scaling the Teeth* injures them is unfounded.

"The tartar is nothing more than a calcareous secretion that impregnates the saliva, and if a nucleus is allowed to form, it rapidly collects on the teeth. It should be removed by the operation of scaling, which may be performed without endangering the teeth at all. Some persons suppose that removing it destroys them; similar, we presume, to the French, who suppose that combing children's hair is unhealthy. A distinction should be made between the tartar and a discoloration that sometimes settles round the necks of the teeth. If this latter is scraped away with the instruments too roughly, it may injure the enamel, but not so the removal of the tartar. Tartar does not decay the teeth, but causes the gums to recede; so that in time the teeth loosen, and fall out. Once a year, and with some persons less frequently, is sufficient to have it removed. A proper attention to cleanliness will, when young, almost always prevent its collection. After a serious illness, the teeth should be always cleaned by a dentist; for, during its continuance, seldom any attention can be paid to them: and the feverish state of the stomach, added to the acids mostly contained in the medicines, will do more harm to them than anything else. But if they are cleaned as soon as the convalescent can support the operation, which *should not* give the least pain, the teeth will grow healthy again with the rest of the body."

We will conclude our notice of this little work with an extract concerning *Tooth-powders.*

"Which is the best powder? This would be a very difficult task to decide. Most dentists prepare it; and it is reasonable to suppose that a dentist of reputation will not give anything likely to be injurious to the teeth, and, provided the powder contain *no acid,* and is quite impalpable, it seldom does harm. But if I cannot name which is the best, I shall at all events name two of the very worst, viz. soot and charcoal. 'What!' it will be exclaimed, 'soot and charcoal not good! Why, look at the chimney-sweeps, in support of the former; and almost everybody recommends the latter.' I shall simply observe, as to the first, that what looks beautifully white in a sooty-faced chimney-sweeper, would look passably yellow and dirty in a young lady of eighteen. It is the contrast that makes the teeth appear so white and the gums so ruddy. Soot contains a considerable quantity of acid, and is on that score bad. I have moreover examined several chimney-sweepers' mouths, which were far from being healthy. With respect to charcoal, and all calcined substances of the same nature, they are decidedly objectionable. Charcoal has, from its antiseptic properties, the peculiar power of drawing to itself all extraneous matter; so that anything in a putrid state being surrounded by it will be divested of its fætid matter, which is transferred to the charcoal. This property would therefore make it a very valuable article, if cleaning the teeth consisted merely in putting a piece of it into the mouth, and there allowing it to be stationary; but, being reduced to a powder, and rubbed on the teeth, some of the particles must get between them, or lodge where decay may have commenced, and thus form a nucleus for collecting extraneous substances, instead of neutralizing them. If we could be sure that no particle of charcoal would remain in the interstices of the teeth, after we had rinsed the mouth, it would not be objectionable; but, as that is impossible, a substance which has so dangerous a tendency, ought to be discarded. Let those persons who are in the habit of using these powders examine their teeth, and they will find that there is a blueish black appearance between them that is not natural. It does not however follow, that all those persons who have been in the habit of using charcoal must necessarily have bad teeth: it may tend to decay them, and that is sufficient for me to reject it: added to which, it is dirty."

The volume is terminated by an essay on Artificial Teeth, which those in want of these useful implements will do well to consult.

Mr. O'Shaugnessy has published his pamphlet upon Tumours of the Jaws, requiring excision or extirpation, especially for the instruction of the students and graduates of the Bengal Medical College. Although the removal of the whole or part of the superior or inferior maxilla is now recognized as a legitimate surgical operation, the author seems to us, considering he is addressing young men, not to have dwelt sufficiently upon its formidable nature, and the rarity of the cases in which it can with propriety be performed. When we know that experienced hospital-surgeons have, dazzled by the *eclat* consequent upon brilliant surgical achievements, undertaken it in cases wherein the speedy re-appearance of the disease exposed their error almost before the praises bestowed upon them had died upon their ears; and when we consider the acknowledged difficulty of determining the malignity of some of the tumours affecting these parts, we think more cautionary observations should have been addressed. When, too, we know with what care some of our most employed surgeons have refreshed their knowledge of the parts implicated in operations they were about to undertake, we think the injunction "I would therefore recommend the young surgeon, before proceeding to this operation, to refresh his recollection of the anatomy of this region, *practically if possible*," is worded far too mildly. Here is no pressing urgency, as in hæmorrhage or in hernia, and let no man venture upon this formidable procedure without having again and again examined the parts just prior to commencing it.

The diseases enumerated and briefly described by Mr. O'Shaugnessy as those which justify an operation, are Fibro-cartilaginous Tumours, the benign form of Osteo-sarcoma, Spina Ventosa, and Exostosis. Besides these are other affections of a less formidable character, and requiring less extensive interference, as Epulis, or solid growth from the gums and periosteum, Exfoliation, Abscess of the Antrum, and Abscess at the side of the lower Jaw. Upon the last-mentioned affection there is the following observation.

"It sometimes happens that an abscess forms on the side of the inferior maxilla, either internally, between the cheek and gums, or externally, near the symphysis of the chin, or on the side of the bone close to its angle. This is at first frequently taken to be a common collection of matter, which, when opened, and emptied, is expected to heal in the usual way, but a sinus forms, and continues to discharge through a fistulous opening. If the patient happens to apply to a medical man who is ignorant of the cause of his disease, the chances are he is subjected to a variety of treatment, for scrofula, caries of the jaw, &c. &c. when by merely extracting a tooth it might be cured in a few days. The crowded state of the teeth causes inflammation in the periosteum and sockets of these bodies, and until room is made by extracting one of them, the discharge continues. These abscesses generally form in persons from the age of 19 to 24, when the wisdom teeth are coming forward. It is not in general of much consequence which of the teeth in the neighbourhood of the fistula is extracted, but whether the teeth are sound, or unsound, one must be taken out before the discharge of matter ceases, which it is usually found to do in a day or two after the tooth is drawn, although it may have existed for years previously."

The surgical anatomy of the jaws and the various stages of the operation are clearly described. Five cases in which it was successfully performed are also given. Four of these consisted of osteo-sarcoma and one of spina-ventosa. In two, the superior maxilla was completely, and in one, partially extirpated. In one of the remaining cases a part, and in the other the whole, of the lower maxilla was removed. They form a valuable contribution to this branch of surgery, and do great credit to the surgical skill of the narrator.

